# Exploring the diverse factors influencing healthcare utilization during the COVID-19 crisis

**DOI:** 10.3389/fpubh.2025.1512735

**Published:** 2025-05-16

**Authors:** Bhumi Chaturvedi, Preeti Raj, Pratima Singh, Hemant Bareth, Anupama Sharma, Mahaveer Singh, Deepak Nathiya, Balvir Singh Tomar

**Affiliations:** ^1^Department of Pharmacy Practice, Nims Institute of Pharmacy, Nims University Rajasthan, Jaipur, India; ^2^School of Health Sciences, Faculty of Biology, Medicine and Health, Manchester Academic Health Science Centre, University of Manchester, Manchester, United Kingdom; ^3^Public Health and Preventive Medicine, University of Alberta, Edmonton, AB, Canada; ^4^Department of Biochemistry, National Institute of Medical Sciences and Research, Nims University Rajasthan, Jaipur, India; ^5^Department of Endocrinology, National Institute of Medical Sciences and Research Nims University Rajasthan, Jaipur, India; ^6^Department of Clinical Studies, Fourth Hospital of Yulin (Xingyuan), Yulin, Shaanxi, China; ^7^Department of Clinical Sciences, Shenmu Hospital, Shenmu, Shaanxi, China; ^8^Institute of Pediatric Gastroenterology and Hepatology, National Institute of Medical Sciences and Research, Nims University Rajasthan, Jaipur, India

**Keywords:** COVID-19, healthcare utilization, length of hospital stay, pandemic, SARS-CoV-2

## Abstract

**Background:**

The emergence of the COVID-19 pandemic created an unprecedented global health crisis, resulting in major disruptions to healthcare systems worldwide. The pandemic has also significantly reshaped healthcare utilization patterns. This study aimed to assess healthcare utilization during the three waves of COVID-19.

**Methods:**

We conducted a retrospective study involving 1,308 patients admitted to the COVID-19 care facility at the National Institute of Medical Sciences and Research in Jaipur, Rajasthan, India. The study analyzed healthcare utilization patterns during the first, second, and third waves of COVID-19, focusing on patient hospitalization duration across the three waves.

**Results:**

The hospitalization rate increased during the second wave compared to the first wave and subsequently declined in the third wave. Hospitalization durations varied significantly across the waves. In all three waves, 30% of the population was hospitalized for 0–5 days, 25.9% for 9–13 days, 24.08% for 6–8 days, and 19.5% of patients were hospitalized for more than 14 days. A *p*-value of 0.032 indicated a statistically significant difference in length of hospital stay (LHS) across the three waves of COVID-19. A threshold p-value of 0.05 was used to assess healthcare utilization and to estimate future healthcare requirements for similar pandemic scenarios.

**Conclusion:**

Our findings highlight the dynamic nature of healthcare demands during pandemic waves and underscore the need for flexible healthcare systems capable of adapting to fluctuating patient loads. Proactive planning and resource allocation are crucial to managing future pandemics effectively.

## Introduction

The COVID-19 pandemic has had a profound impact on healthcare systems globally. The initial outbreak was first detected in Wuhan, China. As the virus’s behavior in China was studied, other countries began preparing for the impending waves of the pandemic. However, many nations, including India, lacked sufficient time to prepare adequately. In response to the pandemic’s waves, India implemented various healthcare and treatment strategies (1–3).

During the first wave, the rapid spread of the virus, coupled with uncertainty about treatment and outcomes, put immense pressure on India’s healthcare system (4). Hospitals prioritized COVID-19 care to manage the surge in patients, while non-essential medical procedures were postponed or avoided by patients (5).

The second wave brought additional challenges, severely disrupting healthcare services across the country. Shortages of medical supplies, an inadequate number of healthcare personnel, and overwhelmed hospitals led to increased mortality and morbidity rates from COVID-19 (6). In response, hospitals began streamlining resources and implementing standard operating procedures. This allowed the country to address healthcare challenges more effectively by expanding local facilities, advancing telemedicine, and integrating e-health services (7, 8).

With the onset of the third wave, the emergence of new SARS-CoV-2 variants introduced further complexities in healthcare management. The availability and distribution of vaccines, alongside vaccine hesitancy, significantly influenced healthcare decisions. The evolution of additional variants highlighted the importance of timely and appropriate care-seeking behaviors (9, 10).

To date, no comprehensive study has compared healthcare utilization across the three waves of COVID-19 in terms of hospital stay durations, admission units, and medication regimens. Such research is crucial to understanding the full burden of the pandemic and providing insights that can improve healthcare preparedness for future crises.

This study aims to analyze the factors influencing healthcare utilization patterns during the three waves of COVID-19 in India, with a focus on hospital admissions, and the determinants of Health Care Utilization (HCU). With this, a clearer understanding of the pandemic’s impact on hospital admissions and guide improvements in future healthcare responses.

## Methodology

### Study design

The acute COVID care unit of National Institute of Medical Sciences and Research, Nims Hospital Rajasthan, Jaipur, India admitted 1,620 COVID-19 patients over the three waves of pandemic. We included data of the patients admitted during the first, second, and third waves.

### Data collection

A retrospective analysis was performed to investigate the healthcare utilization of hospitalized COVID-19 patients during the first wave (March–November 2020), the second wave (March–May 2021), and the third wave (January–February 2022) of the pandemic (11). This assessment was carried out utilizing records from the Medical Record Department (MRD) of the Nims Hospital, Rajasthan, Jaipur, India. The Nims hospital admitted 1,000 RT-PCR-positive patients in the first wave, 597 in the second wave, and only 23 in the third wave. We extracted medical information from 1,620 patients and assessed them for research inclusion and exclusion criteria. Of them, we found 1,308 to be eligible for the study. A total of 42 (3.21%) admitted patients had missing dataset, participants with missing values were excluded entirely from the analysis.

All symptomatic and asymptomatic patients with a positive RT-PCR report were included in the study, while patients with incomplete data and those who died during their hospital stay were excluded.

The retrospective data for these patients were acquired using a data collecting form and then entered into a Microsoft Excel spreadsheet. The data included demographic information such as age, gender, and address type (urban versus rural), as well as clinical information such as duration of hospital stay (LHS), High-resolution computed tomography (HRCT) score categorized into mild (<8), moderate (9–15), and severe levels (16–25) (12), Admission unit including ICU, CCU, General ward, Deluxe and super deluxe and Isolation ward, Comorbidities such as diabetes, hypertension, dyslipidemia, respiratory disease were documented based on the patient history, treatments including Corticosteroids, Hydroxychloroquine, Monoclonal antibodies, and Antiviral drugs such as Remdesivir, Favipiravir, Molnupiravir, Daclizumab, Bevacizumab. The age of patient population were grouped into 5 and length of hospital (LHS) stay into 4 categories (refer Tables 1, 2).

**Table 1:** Demographic characteristics of 1,308 patients diagnosed with COVID-19 across all the three waves.

Parameter	Overall (1308)	I wave (867)	II wave (418)	III wave (23)	*p*-value^*^
Gender					
Male	904 (69.11)	631 (72.77)	265 (63.39)	8 (34.78)	
Female	404 (30.88)	236 (27.22)	153 (36.60)	15 (65.21)	
Other	0 (0.00)	0 (0.00)	0 (0.00)	0 (0.00)	**<0.001** ^*^
Age (years)					
0–20	40 (3)	27 (3.11)	7 (1.67)	6 (26.08) 6 (26.08)	**0.016** ^**^
21–40	428 (32.72)	283 (32.64)	139 (33.25)	5 (21.73)	
41–60	483 (36.92)	290 (33.44)	188 (44.97)	5 (21.73)	
61–80	342 (26.14)	253 (29.18)	84 (20.09)	1 (4.34)	
>80	15 (1.14)	14 (1.61)	0 (0.00)	6 (26.08) 6 (26.08)	
Address					
Rural	905 (69.18)	612 (70.58)	273 (65.31)	20 (86.95)	**0.027** ^*^
Urban	403 (30.81)	255 (29.41)	145 (34.68)	3 (13.04)	
LHS					
0–5	398 (30.42)	264 (30.44)	119 (28.46)	15 (65.21)	**<0.001** ^*^
6–8	315 (24.08)	222 (25.60)	88 (21.05)	5 (21.73)	
9–13	339 (25.91)	237 (27.33)	100 (23.93)	2 (21.73)	
>14	256 (19.57)	144 (16.60)	111 (26.55)	1 (4.34)	

All the data are presented in numbers and percentage (%) calculated within each subgroup (e.g., gender distribution within each wave).

The *p*-values presented in the table were calculated using the Chi-square test and Fisher’s exact test, depending on the suitability based on cell counts. These *p*-values indicate statistically significant differences in the distribution of variables across the three waves (I Wave, II Wave, and III Wave), significant values are marked in bold.

LHS, Length of Hospital stay (days).

**Table 2:** Clinical parameters influencing the utilization of the healthcare resources.

Parameter	Overall (1308)	I wave (867)	II wave (418)	III wave (23)	*p*-value*
HRCT score					
Mild (<8)	232 (17.73)	161 (18.56)	67 (16.02)	6 (26.08)	**<0.001**
Moderate (9–15)	382 (29.20)	285 (32.87)	93 (22.24)	7 (30.43)	
Severe (16–25)	694 (53.05)	421 (48.55)	258 (61.72)	10 (43.47)	
Admission unit					
ICU	83 (6.34)	5 (0.57)	77 (18.42)	1 (4.34)	
CCU	437 (33.40)	288 (33.21)	143 (34.21)	6 (26.08)	
General ward	11 (0.84)		7 (1.67)	1 (4.34)	
Deluxe and super deluxe	514 (39.39)	3 (0.34)	136 (32.53)	2 (8.69)	**<0.001**
Isolation ward	263 (20.10)	376 (43.36) 195 (22.49)	55 (13.15)	13 (56.52)	
Comorbidities					
Diabetes	390 (29.82)	325 (37.48)	61 (14.59)	4 (17.39)	**0.001**
Hypertension	356 (27.22)	265 (30.56)	86 (20.57)	5 (21.74)	**<0.001**
Dyslipidaemia	245 (18.73)	210 (24.22)	32 (7.65)	3 (13.04)	**<0.001**
Respiratory disease	148 (11.31)	118 (13.61)	26 (6.22)	4 (17.39)	**<0.001**
Treatment					
Non-invasive mechanical ventilation	58 (4.43)	18 (2.07)	38 (9.09)	2 (8.69)	**<0.001**
Invasive mechanical ventilation	103 (7.87)	87 (10.03)	16 (3.82)	0 (0)	
High flow oxygen therapy	90 (6.88)	42 (4.84)	44 (10.52)	4 (17.39)	
Conventional oxygen therapy	681 (52.06)	524 (60.43)	148 (35.40)	9 (39.13)	
Corticosteroid	376 (28.74)	196 (22.60)	172 (41.14)	8 (34.78)	
Hydroxychloroquine	813 (62.15)	472 (54.44)	324 (77.51)	17 (73.91)	
Monoclonal antibodies	302 (23.08)	108 (12.45)	187 (44.73)	7 (30.43)	
Antiviral therapy	866 (66.20)	524 (60.43)	324 (77.51)	18 (78.26)	

All the data are presented in numbers and percentage (%) calculated within each subgroup (e.g., gender distribution within each wave).

*p-value was calculated using chi-square test & Fisher exact test. The *p*-values presented in the table were calculated using the Chi-square test and Fisher’s exact test, depending on the suitability based on cell counts. These *p*-values indicate statistically significant differences in the distribution of variables across the three waves (I Wave, II Wave, and III Wave), significant values are mark in bold.

LHS, Length of Hospital stay (days).

### Data analysis

We analyzed the data with IBM SPSS (version 26; IBM, Armonk, NY, USA). We expressed all categorical variables in percentages and analyzed them using the chi-square test or Fisher exact test to identify significant differences between the first, second, and third waves calculated within each subgroup (e.g., gender distribution within each wave). A *p*-value of <0.05 was considered statistically significant. We used Poisson analysis to investigate the relationships between demographic characteristics (age, gender, comorbidities) and the occurrence of COVID-19 over three waves as a dependent variable. Multivariable Poisson regression is a type of generalized linear model (GLM), this model assumes that the dependent variable (COVID-19 occurrence) follows a poisson distribution. Since the study spans three COVID-19 waves, poisson regression is useful in assessing whether the incidence of cases changed significantly over time while adjusting for demographic variables. All graphical representations were made using Microsoft Excel version 2019.

## Results

**Figure 1:**
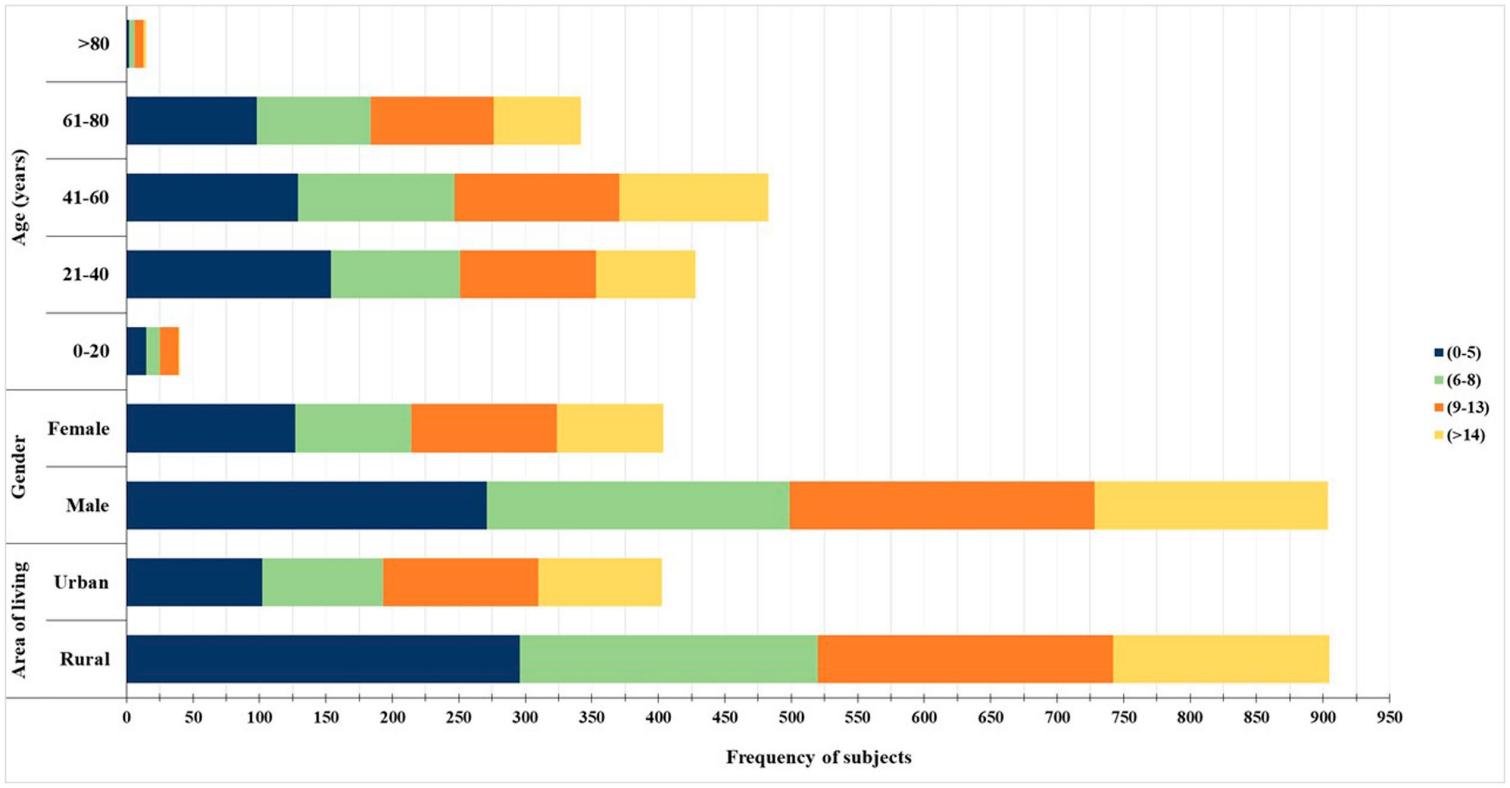
Distribution of healthcare utilization based on age group, gender, and area of residence. The bar chart illustrates the frequency of healthcare utilization among study participants stratified by age (in years), gender (male and female), and area of residence (urban and rural). The duration of healthcare utilization is categorized into four groups: 0–5 days (dark blue), 6–8 days (light green), 9–13 days (orange), and more than 14 days (yellow). Middle-aged individuals (21–60 years), males, and those from rural areas demonstrated longer healthcare usage durations.

**Figure 2:**
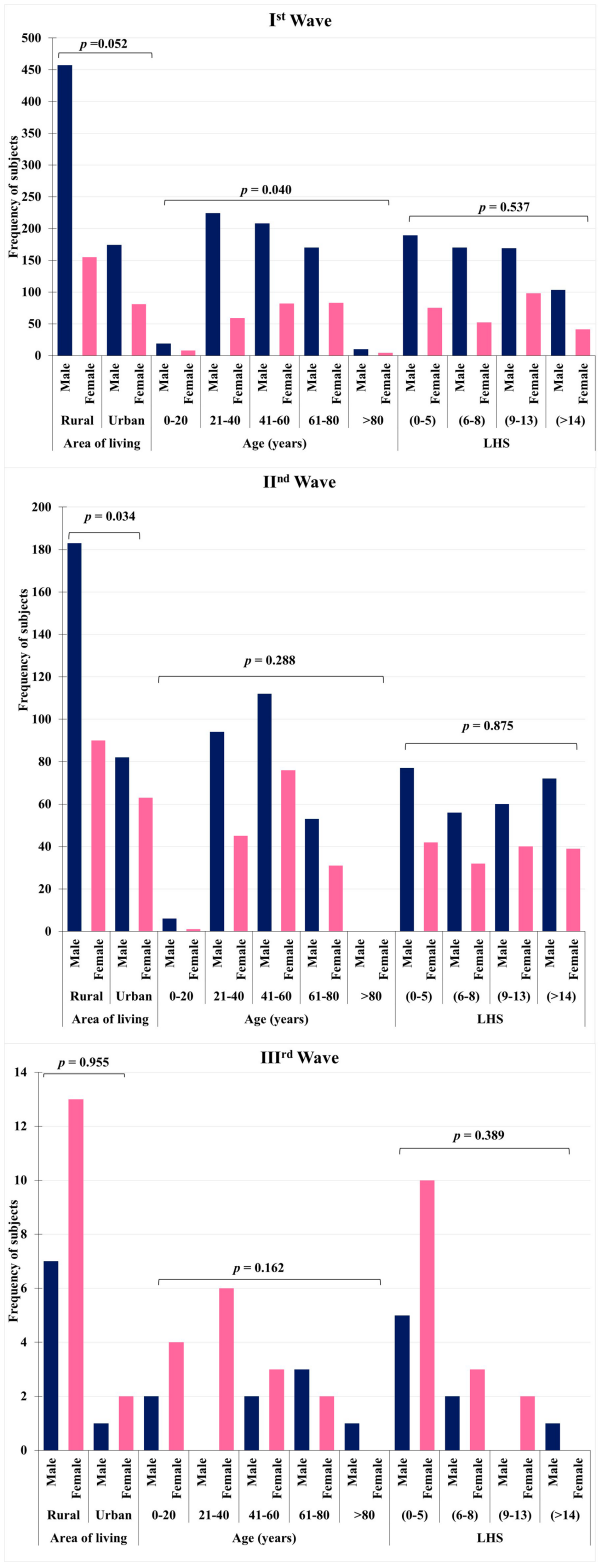
Comparison of healthcare utilization across the three COVID-19 waves based on gender, age, and area of residence. The grouped bar charts display the frequency of healthcare utilization among COVID-19 patients during the first, second, and third waves, categorized by gender, age group, area of residence, and duration of utilization (LHS: length of healthcare stay). Each chart presents statistical comparisons (*p*-values) for gender-based, age-based, and residence-based differences in healthcare usage. The data highlight gender and rural–urban disparities and trends in age-specific healthcare engagement across different waves.

A total of 1,620 cases of COVID 19 were admitted to acute COVID care facility of the National Institute of Medical Sciences and Research, Nims Hospital Rajasthan, Jaipur, India, were categorized based on their occurrence during the first, second, and third waves. Out of 1,620 cases, 1,308 patients were found eligible for the inclusion in this study. Of these 1,308 patients, 867 patients were admitted in the first wave, 418 in the second wave, and 23 cases in the third wave. In this retrospective observational study, we found that the total number of patients admitted subsequently decreased from I wave to III wave. Males to female ratio was 2.23 (*p-*value <0.001) indicating males were more affected than females in all the three waves. The age group most impacted by COVID-19 among the patients were 41–60 year range, which comprises 36.9% of the total. In comparison, 26.14% of the patients were in the 61–80 year range, and only 1.14% of the patients were older than 80 years. This suggests that middle-aged individuals (41–60 years) are more affected by the virus than those who are over 80 years. Patients were divided into four groups as per the length of hospitalization. In these 30% of the patients were hospitalized for 0–5 days, 25.9% for 9–13 days, 24.08% for 6–8 days, and only 19.5% of patients hospitalized for >14 days (refer Table 1). The healthcare utilization data during the COVID-19 pandemic, categorized by area of living, gender, and age groups is shown in Figure 1. Rural residents show higher utilization across all age groups compared to urban counterparts, potentially due to limited access to healthcare in rural areas. Similarly, males and middle age group (41–60 years) utilizes more healthcare resources. Understanding these patterns can inform targeted interventions to address disparities and optimize healthcare delivery during pandemics. The detail description of patients with area of living, age and LHS across all the three waves on the basis of gender has been demonstrated in Figure 2 with the respective *p* values. Table 2 shows hospital burden across pandemic outbreak, the HRCT score indicates that the progression in the number of severe cases from the first to the third wave with more severe cases (61.72%) in second wave, requiring more intensive medical attention and resources. The significant *p*-value (<0.001) suggests a substantial increase in the strain on hospital resources, especially during the second wave, which necessitated more intensive management of severe cases. Admissions to various hospital units such as ICU, CCU, general wards, and isolation wards. 39.39% of the patients were admitted in deluxe and super deluxe ward and 33.40% in critical care unit (CCU) utilizing expensive facilities and treatments, reflecting a greater strain on these critical areas of the hospital. The prevalence of comorbidities among COVID-19 patients, including diabetes 390 (29.82%), hypertension 356 (27.22%), dyslipidemia 245 (18.73%), and respiratory disease 148 (11.31%). Based on the prevalence data provided for comorbidities among COVID-19 patients we can infer that diabetes and hypertension are the most prevalent comorbidities among COVID-19 patients.

The types of treatments administered, ranging from non-invasive methods and high-flow oxygen to specific regimen like corticosteroids, hydroxychloroquine, monoclonal antibodies, and antiviral drugs including Ramdesivir, Favipiravir, Molnupiravir, Daclizumab, Bevacizumab, all these expensive treatment regimens directly influenced the healthcare utilization. The significant values across different categories highlight a clear trend toward increased hospital burden, particularly marked by an increase in severe cases, higher admissions to resource-intensive units, and shifts in treatment strategies. The future trajectory of a pandemic similar to COVID-19 was forecasted by conducting a multivariable poisson regression analysis. The analysis used age, gender, residence (region of living), HRCT, and treatment as independent factors. The results showed a statistically significant relationship male gender (*p* = 0.037; OR 0.90; 95% confidence interval [CI] 0.818 to 0.998), middle age and below (41–60: OR 1.26; 95% CI 0.78–2.06; 0–20: OR; 95% CI 0.78–2.33), rural population (OR 1.01; 95% CI 0.91–1.12), Non-invasive mechanical ventilation + Hydroxychloroquine + Antiviral therapy + Monoclonal antibodies (OR 1.15; 95% CI 0.93–1.43) were associated with higher utilizations of resources. In contrast less hospital stay 9–13 days (OR 0.91; 95% CI 0.79–1.05), HRCT score (mild: OR 0.96; 95% CI 0.84–1.09; moderate: OR 0.93; 95% CI 0.83–1.04) are associated with lower utilization of overall resources.

Other variables, such as age group, vaccination status, comorbidities, and HRCT severity score, did not show statistically significant associations in the multivariate model (*p* > 0.05). However, some variables demonstrated trends that may have clinical relevance. For instance, participants with moderate to severe HRCT scores showed a slightly increased incidence rate ratio, although not reaching statistical significance (adjusted IRR = 1.188; 95% CI: 0.913–1.546; *p* = 0.199) (refer Table 3).

**Table 3:** Multivariable poisson regression analysis of factors associated with COVID-19.

Parameter (*n* = 1,308)	*β*/OR	95% wald confidence interval		*p*-value
		Lower	Upper	
Gender				**0.037**
Male	0.90	0.81	0.99	
Female (ref)	ref	ref	ref	
Age (years)				0.181
0–20	1.35	0.78	2.33	
21–40	1.22	0.75	1.99	
41–60	1.26	0.78	2.06	
61–80	1.16	0.71	1.89	
>80 (ref)	ref	ref	ref	
Address				0.971
Rural	1.01	0.91	1.12	
Urban (ref)	ref	ref	ref	
LHS				0.637
0–5	0.96	0.84	1.10	
6–8	0.92	0.80	1.06	
9–13	0.91	0.79	1.05	
>14 (ref)	ref	ref	ref	
HRCT				0.152
Mild	0.96	0.84	1.09	
Moderate	0.93	0.83	1.04	
Severe	ref	ref	ref	
Treatment				0.977
Treatment 1	1.15	0.93	1.43	
Treatment 2	0.79	0.65	0.97	
Treatment 3	1.07	0.88	1.29	
Treatment 4	0.83	0.74	0.93	
Treatment 5	ref	ref	ref	

Treatment 1 (Non-invasive mechanical ventilation + Hydroxychloroquine + Antiviral therapy + Monoclonal antibodies).

Treatment 2 (Invasive mechanical ventilation+ Hydroxychloroquine + Antiviral therapy+ Monoclonal antibodies).

Treatment 3 (High flow oxygen therapy + Hydroxychloroquine + Antiviral therapy + Monoclonal antibodies).

Treatment 4 (Conventional oxygen therapy + Hydroxychloroquine + Antiviral therapy + Monoclonal antibodies).

Treatment 5 (Corticosteroids + Hydroxychloroquine + Antiviral therapy+ Monoclonal antibodies).

LHS, Length of Hospital stay (days).

Significant values are marked in bold.

## Discussion

In this study, we examined healthcare utilization patterns among COVID-19 patients admitted to the acute COVID care center at NIMS Hospital, Jaipur, Rajasthan, India. A total of 1,308 individuals were included, and their utilization patterns were analyzed based on length of hospital stay, treatment protocols, and the hospital units where they were admitted. During the second wave, a higher number of patients required admission to the ICU and CCU compared to isolation and general wards, indicating greater severity and mortality during this wave. This surge in severity led to a shortage of essential medical supplies, including medications, oxygen, hospital beds, and healthcare personnel. Similar studies have shown that the second wave was more widespread and severe, whereas the first wave was marked by stringent social distancing measures, national lockdowns, and travel restrictions (13–15). Consequently, the strain on healthcare resources during the COVID-19 pandemic exceeded that experienced during recent pandemics involving influenza and other coronaviruses (16).

In the second wave, 26.55% of patients were hospitalized for more than 14 days, compared to 16.60% in the first wave and 4.34% in the third wave. The significant difference in hospital stay duration (*p* < 0.001) highlights the increased severity of illness and higher resource utilization during the second wave. A study by Tendulkar P et al. and Singh S et al. similarly reported that the average hospital stay was longer during the second wave compared to the first (17, 18). Study also demonstrated a marked decline in the number of infected patients during the third wave when compared to the first and second waves. This trend suggests a potential shift in transmission dynamics or the impact of increased immunity and public health interventions over time.

During the second wave, treatments such as Hydroxychloroquine, Monoclonal antibodies, Antiviral therapy, and high-flow oxygen therapy were used more frequently than in the first and third waves. The use of drugs like Remdesivir and Bevacizumab was found to be more effective in preventing disease progression and reducing hospital stay durations (19–21).

In both the first and second waves, the majority of COVID-19 patients were from rural areas, with a *p*-value of 0.028, corroborating findings from Cuadros DF et al. Rural populations often face challenges such as limited access to healthcare resources, leading to delayed diagnoses and treatment, which in turn results in more severe cases and longer hospital stays. Factors such as lack of knowledge about social distancing, limited availability or use of face masks, and delayed vaccination uptake may have contributed to this trend (8, 22). In contrast, a study by Bhocal U. et al. claimed that rural populations had fewer infections and shorter hospital stays due to a stronger immune response, presenting contrary results (23). This study’s significant strengths include its large and diverse patient cohort, along with its comprehensive evaluation of healthcare utilization following a COVID-19 diagnosis.

Several studies have shown that males were more susceptible to COVID-19 compared to females, resulting in higher hospitalization and healthcare resource utilization among men. In our study, 904 males (69.11% of the total) were infected, possibly due to biological differences in immune responses and pre-existing health conditions (24–27), both of which may contribute to higher rates of hospitalization (28). Additionally, the middle-aged population was more affected by COVID-19 than pediatric and geriatric populations in this study (27, 29, 30). In comparison with the findings of the study by Gunjan Kumar et al., which analyzed data from 31 hospitals across India, our study similarly observes a higher proportion of middle-aged individuals affected by post-COVID sequelae. The broader dataset reported by Kumar et al. reinforces our findings, indicating that the middle-aged population represents a significant demographic among COVID-19 survivors experiencing long-term health impacts (30).

This could be attributed to the fact that vulnerable groups, such as children and the older adult, were often kept under strict preventive measures, despite being more immunosensitive (25). Underlying health conditions, such as obesity, diabetes, hypertension, and respiratory diseases, which were prevalent in our study, can exacerbate the severity of COVID-19. This underscores the importance of managing comorbidities in reducing the impact of the virus (31, 32). Although the older adult may have a higher risk of mortality from COVID-19, preventive measures like shielding and vaccination campaigns may have reduced their infection rates compared to other age groups (33, 34).

To the best of our knowledge, this is the first study to forecast future pandemic trends similar to COVID-19 while comparing healthcare utilization patterns across the three waves of the COVID-19 outbreak, based on hospital stay length, admission unit, and treatment regimen in a tertiary care hospital in Jaipur, Rajasthan, India.

### Limitations

Further study of healthcare utilization in multicenter involving bed charge, food, lack of transportation, shortage of manpower, poor cooperation from beneficiaries, details about the health care card are required to find further trend of utilization pattern.

## Conclusion

The healthcare utilization pattern observed during three waves reveals notable shifts. The initial wave witnessed heightened healthcare utilization, primarily driven by increased hospitalizations. The second wave displayed a further surge, indicating evolving patient needs. However, the third wave demonstrated a decline, possibly reflecting enhanced preventive measures. These trends emphasize the importance of adaptable healthcare systems to accommodate varying demands while maintaining a proactive approach to healthcare management. Therefore, any future similar pandemic situation will be more dangerous for females, middle aged population, individuals residing in rural population and will stay for a long period of time in the hospital.

## Data Availability

The original contributions presented in the study are included in the article/supplementary material, further inquiries can be directed to the corresponding author.

## References

[1] Cheng ZJ Zhan Z Xue M Zheng P Lyu J Ma J. Public health measures and the control of COVID-19 in China. Clin Rev Allergy Immunol. (2021) 64:1–6. doi: 10.1007/s12016-021-08900-2 34536214 PMC8449219

[2] Faruqui N Raman VR Shiv J Chaturvedi S Muzumdar M Prasad V. Informal collectives and access to healthcare during India’s COVID-19 second wave crisis. BMJ Glob Health. (2021) 6:e006731. doi: 10.1136/bmjgh-2021-006731 PMC8282415 34257140

[3] Zhu H Wei L Niu P. The novel coronavirus outbreak in Wuhan, China. Global Health Res Policy. (2020) 5:1–3. doi: 10.1186/s41256-020-00135-6 PMC7050114 32226823

[4] Guan WJ Ni ZY Hu Y Liang WH Ou CQ He JX. Clinical characteristics of coronavirus disease 2019 in China. N Engl J Med. (2020) 382:1708–20. doi: 10.1056/NEJMoa2002032 32109013 PMC7092819

[5] Hollander JE Carr BG. Virtually perfect? Telemedicine for COVID-19. N Engl J Med. (2020) 382:1679–81. doi: 10.1056/NEJMp2003539, PMID: 32160451

[6] Wosik J Fudim M Cameron B Gellad ZF Cho A Phinney D. Telehealth transformation: COVID-19 and the rise of virtual care. J Am Med Inform Assoc. (2020) 27:957–62. doi: 10.1093/jamia/ocaa067, PMID: 32311034 PMC7188147

[7] Bouabida K Lebouché B Pomey MP. Telehealth and COVID-19 pandemic: an overview of the telehealth use, advantages, challenges, and opportunities during COVID-19 pandemic. Healthcare. (2022) 10:2293. doi: 10.3390/healthcare10112293 36421617 PMC9690761

[8] Oduro MS Peprah P Morgan AK Agyemang-Duah W. Staying in or out? COVID-19-induced healthcare utilization avoidance and associated socio-demographic factors in rural India. BMC Public Health. (2023) 23:1439. doi: 10.1186/s12889-023-16282-7, PMID: 37501140 PMC10375657

[9] Adams JG Walls RM. Supporting the health care workforce during the COVID-19 global epidemic. JAMA. (2020) 323:1439–40. doi: 10.1001/jama.2020.3972, PMID: 32163102

[10] Gómez CE Perdiguero B Esteban M. Emerging SARS-CoV-2 variants and impact in global vaccination programs against SARS-CoV-2/COVID-19. Vaccine. (2021) 9:243. doi: 10.3390/vaccines9030243 PMC7999234 33799505

[11] Zirpe KG Dixit S Kulkarni AP Pandit RA Ranganathan P Prasad S. The second-vs first-wave COVID-19: more of the same or a lot worse? A comparison of mortality between the two waves in patients admitted to intensive care units in nine hospitals in Western Maharashtra. Indian J Crit Care Med. (2021) 25:1343–8. doi: 10.5005/jp-journals-10071-24042, PMID: 35027792 PMC8693103

[12] Sharma S Aggarwal A Sharma RK Patras E Singhal A. Correlation of chest CT severity score with clinical parameters in COVID-19 pulmonary disease in a tertiary care hospital in Delhi during the pandemic period. Egypt J Radiol Nucl Med. (2022) 53:166. doi: 10.1186/s43055-022-00832-x

[13] Sharma S Singh L Yadav J Gupta U Singh KJ Rao MV. Impact of COVID-19 on utilization of maternal and child health services in India: health management information system data analysis. Clin Epidemiol Global Health. (2023) 21:101285. doi: 10.1016/j.cegh.2023.101285 PMC10063524 37064822

[14] Bager P Wohlfahrt J Rasmussen M Albertsen M Krause TG. Hospitalisation associated with SARS-CoV-2 delta variant in Denmark. Lancet Infect Dis. (2021) 21:1351. doi: 10.1016/S1473-3099(21)00580-6 PMC8415919 34487704

[15] Twohig KA Nyberg T Zaidi A Thelwall S Sinnathamby MA Aliabadi S. Hospital admission and emergency care attendance risk for SARS-CoV-2 delta (B. 1.617. 2) compared with alpha (B. 1.1. 7) variants of concern: a cohort study. Lancet Infect Dis. (2022) 22:35–42. doi: 10.1016/S1473-3099(21)00475-8 34461056 PMC8397301

[16] Huang BZ Creekmur B Yoo MS Broder B Sharp AL. Healthcare utilization among patients diagnosed with COVID-19 in a large integrated health system. J Gen Intern Med. (2022) 37:830–7. doi: 10.1007/s11606-021-07139-z, PMID: 34993879 PMC8735886

[17] Tendulkar P Pandey P Panda PK Bhadoria AS Kulshreshtha P Mishra M. Comparative study between the first and second wave of COVID-19 deaths in India: a single center study. Cureus. (2023) 15:e0248029. doi: 10.7759/cureus.37472 PMC10176533 37187656

[18] Singh S Sharma A Gupta A Joshi M Aggarwal A Soni N. Demographic comparison of the first, second and third waves of COVID-19 in a tertiary care hospital at Jaipur, India. Lung India. (2022) 39:525–31. doi: 10.4103/lungindia.lungindia_265_22, PMID: 36629231 PMC9746281

[19] Costanzo M De Giglio MA Roviello GN. SARS-CoV-2: recent reports on antiviral therapies based on lopinavir/ritonavir, darunavir/umifenovir, hydroxychloroquine, remdesivir, favipiravir and other drugs for the treatment of the new coronavirus. Curr Med Chem. (2020) 27:4536–41. doi: 10.2174/0929867327666200416131117, PMID: 32297571

[20] Horby PW Mafham M Bell JL Linsell L Staplin N Emberson J. Lopinavir–ritonavir in patients admitted to hospital with COVID-19 (RECOVERY): a randomised, controlled, open-label, platform trial. Lancet. (2020) 396:1345–52. doi: 10.1016/S0140-6736(20)32013-4 33031764 PMC7535623

[21] Lam S Lombardi A Ouanounou A. COVID-19: a review of the proposed pharmacological treatments. Eur J Pharmacol. (2020) 886:173451. doi: 10.1016/j.ejphar.2020.173451 32768505 PMC7406477

[22] Cuadros DF Branscum AJ Mukandavire Z Miller FD MacKinnon N. Dynamics of the COVID-19 epidemic in urban and rural areas in the United States. Ann Epidemiol. (2021) 59:16–20. doi: 10.1016/j.annepidem.2021.04.007, PMID: 33894385 PMC8061094

[23] Bhocal U Katyal A Dhull D Raghuraman K Nandal N Gill PS. Assessment of clinical and virological outcomes of rural and urban populations: COVID-19. J Family Med Prim Care. (2022) 11:6074–80. doi: 10.4103/jfmpc.jfmpc_151_22, PMID: 36618254 PMC9810966

[24] Kushwaha S Khanna P Rajagopal V Kiran T. Biological attributes of age and gender variations in Indian COVID-19 cases: a retrospective data analysis. Clin Epidemiol Global Health. (2021) 11:100788. doi: 10.1016/j.cegh.2021.100788 PMC8159626 34079918

[25] Samanta T Gopalan K Devi T. Blocked by gender: disparities in COVID19 infection detection in Tamil Nadu, India. Front Public Health. (2022) 10:966490. doi: 10.3389/fpubh.2022.966490 36249186 PMC9561920

[26] Salerno S Sun Y Morris EL He X Li Y Pan Z. Comprehensive evaluation of COVID-19 patient short- and long-term outcomes: disparities in healthcare utilization and post-hospitalization outcomes. PLoS One. (2021) 16:e0258278. doi: 10.1371/journal.pone.0258278, PMID: 34614008 PMC8494298

[27] Matsunaga N Hayakawa K Asai Y Tsuzuki S Terada M Suzuki S. Clinical characteristics of the first three waves of hospitalised patients with COVID-19 in Japan prior to the widespread use of vaccination: a nationwide observational study. Lancet Reg Health Western Pac. (2022) 22:100421. doi: 10.1016/j.lanwpc.2022.100421, PMID: 35300186 PMC8923875

[28] Bwire GM. Coronavirus: why men are more vulnerable to COVID-19 than women? SN Comprehen Clin Med. (2020) 2:874–6. doi: 10.1007/s42399-020-00341-w PMC7271824 32838138

[29] Kapoor M Nidhi Kaur K Saeed S Shannawaz M Chandra A. Impact of COVID-19 on healthcare system in India: a systematic review. J Public Health Res. (2023) 12:22799036231186349. doi: 10.1177/22799036231186349, PMID: 37461400 PMC10345816

[30] Kumar G Bhalla A Mukherjee A Turuk A Talukdar A Mukherjee S. Post COVID sequelae among COVID-19 survivors: insights from the Indian National Clinical Registry for COVID-19. BMJ Glob Health. (2023) 8:e012245. doi: 10.1136/bmjgh-2023-012245, PMID: 37816536 PMC10565174

[31] Hamza A Shah NN Azad AM Ghanshyam OS Khan Z. Impact of age, gender and comorbidities affecting the severity of COVID-19 infection in Kashmir. J Family Med Prim Care. (2022) 11:1519–24. doi: 10.4103/jfmpc.jfmpc_278_21, PMID: 35516702 PMC9067186

[32] Iftimie S López-Azcona AF Vallverdú I Hernández-Flix S de Febrer G Parra S. First and second waves of coronavirus disease-19: a comparative study in hospitalized patients in Reus, Spain. PLoS One. (2021) 16:e0248029. doi: 10.1371/journal.pone.0248029, PMID: 33788866 PMC8011765

[33] Liu JQ Xu JW Sun CY Wang JN Wang XT Chen X. Age-stratified analysis of SARS-CoV-2 infection and case fatality rate in China, Italy, and South Korea. Eur Rev Med Pharmacol Sci. (2020) 24:12575–8. doi: 10.26355/eurrev_202012_24054 33336777

[34] Cocuzzo B Wrench A O’Malley C. Effects of COVID-19 on older adults: physical, mental, emotional, social, and financial problems seen and unseen. Cureus. (2022) 14:e29493. doi: 10.7759/cureus.29493 36299954 PMC9588279

